# Unveiling the thioredoxin fold: a systematic review and bioinformatic analysis of protein disulfide isomerase and Dsb family proteins

**DOI:** 10.3389/fbinf.2026.1776111

**Published:** 2026-04-07

**Authors:** Daniel Cuevas Ortiz, Karen Werner, Maria Fernanda Frias Mayo, Pablo A. Cárdenas Arredondo, Gabriela F. García Manzano, Cristina Revilla-Monsalve, Héctor Retana, Nelly F. Altamirano-Bustamante, Myriam M. Altamirano-Bustamante

**Affiliations:** 1 Unidad de Investigación en Enfermedades Metabólicas, Centro Médico Nacional Siglo XXI, IMSS, Ciudad de México, Mexico; 2 Servicio de Endocrinología, Instituto Nacional de Pediatría, Ciudad de México, Mexico

**Keywords:** chaperone activity, Dsb proteins, isomerase activity, protein design, protein disulfide isomerase, protein evolution, redox reactions, thioredoxin

## Abstract

**Introduction:**

Protein Disulfide Isomerases (PDIs) and bacterial Dsb proteins are key members of the thioredoxin-fold superfamily, essential for oxidative protein folding in eukaryotic and prokaryotic systems, respectively. Despite their differences in cellular context, these proteins share a conserved thioredoxin domain architecture that enables catalysis of disulfide bond formation, isomerization, and reduction. This systematic review integrates biochemical, structural, and bioinformatic data to identify conserved features within the PDI and Dsb families that underline their catalytic functions.

**Methods:**

Using a PRISMA-based methodology, we screened and analyzed 96 relevant articles and conducted a comparative structural analysis of 11 representative PDI proteins, most of which lack experimentally resolved structures. We leveraged AlphaFold models alongside crystal structures of canonical PDI (PDIA1), DsbC, and DsbG.

**Results:**

We reveal conserved tertiary folds, catalytic motifs, and domain arrangements across species. These findings highlight the evolutionary conservation and structural versatility of thioredoxin-fold enzymes and underscore their biomedical relevance in diseases linked to protein misfolding, such as neurodegeneration, cancer, and infection.

**Discussion:**

The results offer a foundation for future experimental studies and therapeutic exploration targeting redox-regulating thioredoxin-fold proteins.

## 1 Introduction

Disulfide bonds formed through the oxidation of cysteine thiol groups play a central role in stabilizing protein structure and ensuring proper folding, especially in oxidative cellular compartments like the endoplasmic reticulum (ER) (D’Ambrosio et al., 2006). These bonds are crucial for the stable structure of proteins that travel out of cells central regions in both simple and complex organisms. The process of creating these bonds is not just important for maintaining protein shape (Darby and Creighton, 1995); it also plays a role in how cells communicate and regulate various functions (Appenzeller-Herzog and Ellgaard, 2007). While compounds like glutathione disulfide (GSSG) can facilitate the formation of disulfide bonds, they usually do so quite slowly under normal body conditions (Neves et al., 2017). In living organisms, specialized proteins such as protein disulfide isomerase (PDI) and DsbA, belong to the Dsb protein family, are responsible for speeding up this process. These proteins have special areas, termed thioredoxin-like domains, because they share a common structure with thioredoxin, a protein known to promote reduction reactions (Frech et al., 1996).

Enzymes in the thioredoxin superfamily, despite their varied sequences, show a striking similarity in their three-dimensional structure. They all have a special feature in their active site, which is the part of the enzyme that carries out the chemical reaction. This feature is a specific sequence of amino acids, abbreviated CXXC, where ‘C’ stands for cysteine and ‘X’ can be any amino acid (Frech et al., 1996). In this sequence, the first cysteine is usually ready to react because at the pH found in our bodies, it loses a proton, leaving it negatively charged and exposed. The second cysteine, in contrast, typically has a proton and is less reactive, tucked away inside the enzyme. The exact amino acids that sit between these two cysteines differ among various enzymes, but the position of this important CXXC sequence is consistent, always located at the end of an alpha helix, which is a common shape in protein structures (Palma, 2024).

In the space between the inner and outer layers of prokaryotic cells, known as the periplasm, proteins form disulfide bonds through a system called the Dsb protein system. This system carries out two main tasks that work together, even though they sort of ‘compete’ with each other. One part of the system, made up of proteins DsbA and DsbB, helps to form these disulfide bonds. Another part, involving proteins (Luo and Guan, 2012) DsbC, DsbG and DsbD helps to shuffle these bonds around when they do not form correctly the first time (Palma, 2024). DsbA is a powerful player in this system, quickly adding oxygen to the mix to create bonds in proteins and peptides, a process called cysteine-thiol oxidation. However, DsbA sometimes makes mistakes, creating bonds in the wrong places. When this happens, DsbC steps in to fix these errors by rearranging the bonds into their correct spots. Meanwhile, DsbB is a membrane protein that has the job of recharging DsbA with the ability to form more disulfide bonds (Wang, 2012).

PDI is a protein found in the endoplasmic reticulum of eukaryotic cells and has a molecular weight of approximately 55 kDa. Structurally, PDI is composed of several domains arranged in the sequence abb’a′c, where the a and a′ domains are crucial for its enzymatic function. These domains each contain an active site with the sequence CGHC that alternates between a reduced (thiols) and an oxidized (disulfide) state. This redox cycling allows PDI to facilitate various processes involving disulfide bonds in other proteins, such as creating, rearranging, or breaking these bonds (Westphal et al., 1998). For PDI to successfully transfer disulfide bonds to a protein, it must be in its oxidized form. Conversely, when proteins are incorrectly folded, often having incorrect disulfide linkages, PDI inverses to correct these errors (Kim, 2009). It achieves this by catalyzing isomerization reactions; it breaks the improper disulfide bonds and assists in their reformation into correct native disulfide arrangement, ensuring proper protein folding and function.

The human PDI family comprises over 20 thioredoxin-like proteins, including well-characterized members such as ERp44, ERdj5, ERp72, ERp57, ERp46, ERp27, and P5 (PDIA6), which share structural homology with PDIA1 but exhibit diverse catalytic and regulatory functions within the endoplasmic reticulum (ER). These proteins differ in the number and arrangement of thioredoxin-like domains, presence of redox-active CXXC motifs, and additional features such as ER retention signals or substrate-binding domains. For instance, ERp57 interacts with calnexin/calreticulin and participates in MHC class I assembly, whereas ERdj5 facilitates reductive unfolding *via* J-domain interaction with BiP. ERp44 lacks catalytic activity but regulates redox homeostasis and oligomer formation by retaining unassembled subunits in the ER. This functional diversity enables the PDI family to mediate not only oxidative folding but also quality control, reductive isomerization, and ER-associated degradation. Despite these differences, structural alignment reveals a conserved thioredoxin core fold across family members, underscoring a shared evolutionary origin and mechanistic flexibility. This study focuses primarily on PDIA1 and bacterial Dsb proteins, but the broader insights derived from sequence and structure alignments are relevant to understanding the functional architecture of the entire PDI family.

The role of PDI is crucial in maintaining proteostasis, and its dysfunction has been increasingly implicated in the development of neurodegenerative diseases. In conditions such as Alzheimer’s disease, PDI’s isomerase activity is impaired by S-nitrosylation of its active site cysteines, disrupting its ability to manage protein folding (Uehara et al., 2006). These neurodegenerative conditions are often precipitated by disturbances in the redox homeostasis of the endoplasmic reticulum (ER), particularly in the balance of buffers such as glutathione (GSH/GSSG) and hydrogen peroxide (H_2_O_2_/O_2_). Disruption of these systems can lead to improper protein folding and aggregation due to the exposure and interaction of hydrophobic protein regions. Beyond neurodegeneration, PDI has also emerged as a therapeutic target in cancer, where it is often overexpressed in malignant cells. Selectively targeting PDI in these contexts presents promising opportunities for intervention in proteomic medicine, especially in diseases characterized by redox imbalance and protein misfolding.

**FIGURE 1:**
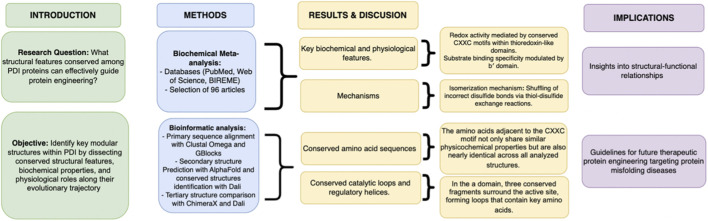
Roadmap of article. This study explores the evolutionary conservation of structural features in PDI (protein disulfide isomerase) proteins to inform strategies in protein engineering. By identifying modular domains and correlating them with biochemical and physiological functions, the research aims to pinpoint key determinants that can guide the rational design of engineered proteins.

This article explores the structural characteristics of Protein Disulfide Isomerase (PDI) proteins that are conserved across species, with the goal of informing protein design strategies for therapeutic applications. Specifically, the research question guiding this study is: *“What are important features to consider in PDI design?”* including relevant amino acids, conserved structures, and activity-related properties (Figure 1). To address this, our objectives were to identify key modular structures within PDI by dissecting conserved structural features, biochemical properties, and physiological roles along their evolutionary trajectory. Our approach began with a systematic review of existing literature to consolidate current knowledge about the biochemical mechanisms and functionalities of PDIs, as well as to highlight gaps in our understanding. Subsequently, we performed a bioinformatic analysis to structurally characterize PDIs and identify conserved regions. The main findings from our study include the identification of five conserved scaffold segments within eleven analyzed PDIs. In a domain, we identified three key conserved regions: the top of the active site (helix 3-loop-beta 4) and two lateral scaffolds (beta 2-loop-helix 2 and loop-helix 4), each containing highly conserved amino acid residues. In a' domain, two additional conserved segments were found: beta 16-loop-helix 12 and beta 17-loop-helix 13. These conserved structural elements represent critical insights from nature that could guide future protein engineering efforts targeting diseases associated with protein misfolding.

## 2 Materials and methods

A systematic review was conducted following PRISMA guidelines and the PICO framework. The research question was: *“What are important features to consider in PDI design?”* including relevant amino acids, conserved structures, and activity-related properties.

Literature research was performed in PubMed, Web of Science, and Bireme between July and August 2024 using combinations of the following terms.

• P (participants): Protein disulfide isomerase (PDI), foldases, DsbA/B/C, thioredoxin-related terms
• I (intervention): Reaction mechanism, catalytic residues, protein interactions, inhibition
• C (comparison): It does not apply.
• O (outcome): Kinetics, catalytic efficiency, binding constants, environmental effects

**FIGURE 2:**
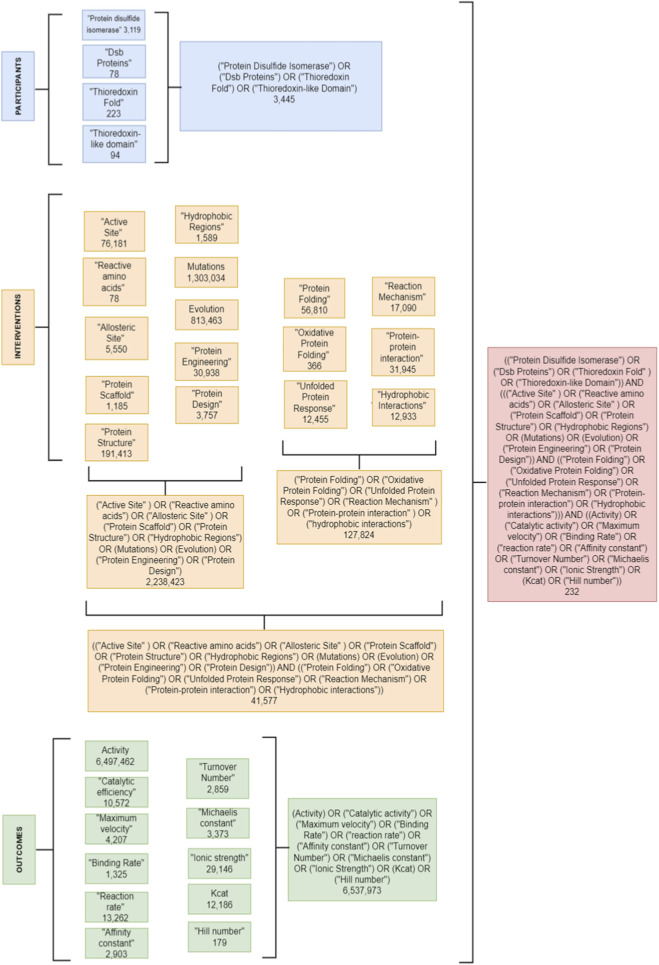
PubMed’s decision tree diagram. This figure describes in full detail the searching strategy in the PubMed database and the number of resulting articles. Subsequently, the results obtained from these searches were downloaded into the Mendeley database. Then, the double references were merged.

Terms were grouped using “OR” within each PICO element and combined using “AND.” The search strategy is detailed in Figure 2. Results were managed in Mendeley and duplicates removed.

Articles were assessed using a 5-criterion quality checklist (objective clarity, research question, methodology, concept definitions, and result alignment), each worth 20 points. Articles scoring ≥80/100 were included.

**FIGURE 3:**
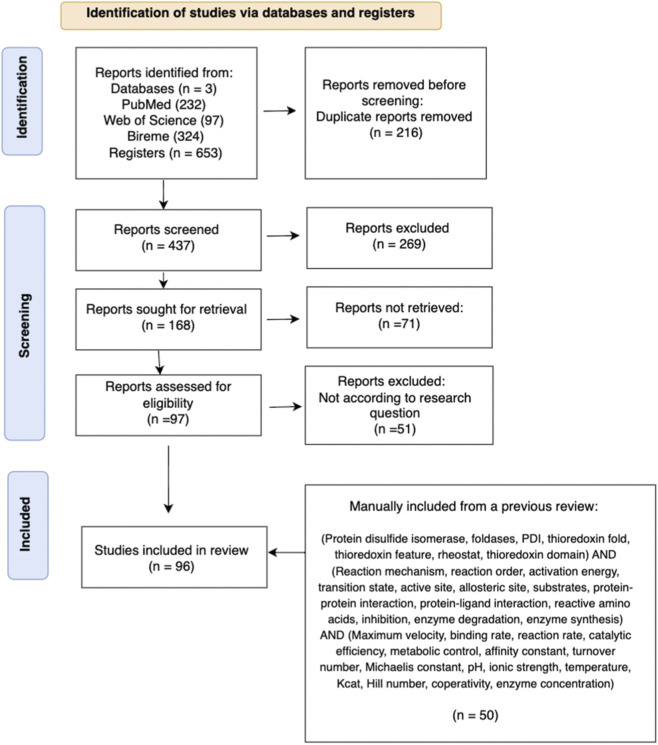
PRISMA flowchart. This flowchart shows the selection and screening process used to obtain the final articles that were included in this paper. Note that the irrelevant topic section refers to topics that were not useful to answer our research questions.

Of 653 articles retrieved, 437 remained after de-duplication. Title screening excluded 269. From 168 full-text attempts, 97 were retrieved, and 46 passed naive reading. An additional 50 relevant articles were included, resulting in 96 final studies (see PRISMA flowchart, Figure 3).

### 2.1 Bioinformatic analysis

#### 2.1.1 Phylogenic and primary structure analysis

A UniProt search retrieved 35 manually reviewed PDIs, filtered for experimental evidence and the CXXC motif. Redundancy (>70% similarity) was reduced using CD-HIT, yielding 11 representative sequences. Phylogenetic analysis was conducted in MEGA (1,000 bootstraps), and alignments performed with Clustal Omega. Conserved blocks were refined using GBlocks and visualized in Jalview. Amino acid conservation was further validated using the Dai tool.

#### 2.1.2 Secondary structure analysis

PDB entry 2B5E (*S. cerevisiae* PDI) and AlphaFold models were aligned using Dali. DsbC and DsbG structures from various bacteria (PDB entries: 1EEJ, 1T3B, 4I5Q, 1V57, 3TGD, 4IHU) were similarly analyzed to determine consensus secondary elements.

#### 2.1.3 Tertiary structure analysis

3D alignments were performed using ChimeraX to identify conserved domains, hydrogen bonds, and hydrophobic regions. Structural comparisons between human PDIA1, DsbC, and DsbG revealed conserved architectural features and functional motifs.

The 29 proteins used to construct the phylogenetic tree were selected from UniProt based on reviewed status, sequence completeness, and the presence of the conserved CXXC motif. From this set, 11 representative proteins spanning major evolutionary clades were chosen to reduce redundancy and ensure broad taxonomic coverage. Of these, 10 lacked experimentally determined structures and were modeled using AlphaFold. Only the structure of *Saccharomyces cerevisiae* PDI was experimentally resolved; all others were obtained through AlphaFold predictions.

## 3 Results

### 3.1 Evolution of the thioredoxin fold

Today proteins are the result of gradual changes from their ancient forms. These changes are recorded in the proteins found across different living species. Thanks to advances in the study of evolutionary relationships and the ability to make DNA in the lab, scientists have been able to recreate genes from very old common ancestors–the shared forebears of bacteria, more complex animals, and vertebrates. Research has found that the general shapes and functions of proteins stay more consistent over time than the exact sequence of their building blocks. Interestingly, these ancient proteins seem to be tougher, better able to withstand heat and chemical reactions. The big question in understanding how proteins have evolved is figuring out how tiny changes in the sequence of amino acids can tweak a protein’s function without changing its overall shape. Further studies reveal that every part of a protein is interconnected, and these connections differ from one protein to another. This network of connections adds a layer of variety to the protein shapes and functions that are essential for life (Modi et al., 2018).

Modi and colleagues have created a method known as the dynamic flexibility index (DFI), which helps to understand how parts of proteins stay the same or change over time. Their method shows that the parts of proteins that do not bend or twist easily tend to remain unchanged over long periods, while the more flexible parts are likely to evolve faster. They also introduced another measure called the dynamic coupling index (DCI), which uncovers how parts of enzymes that work together can affect the enzyme’s activity through a network of interactions, like communication lines to the active site where the main reaction happens. In the evolution of thioredoxins, for example, there’s a balance between stable and flexible regions, with the α3 helix being stiffer and the α4 more flexible in ancient forms of the protein compared to their modern counterparts. Notably, the core part of the protein, the beta sheet, remains mostly unchanged, becoming only slightly more rigid. It is suggested these minor changes happened so that the protein could function in cooler temperatures and a less acidic environment while still maintaining its overall shape (Modi et al., 2018).

Sometimes, as species evolve, certain features that are no longer needed are not eliminated through mutation caused by natural selection; instead, they merely deteriorate, as noted by Gamiz-Arco and colleagues. They discuss that proteins are used to fold without any help, but over time, cells developed more efficient ways to fold proteins. This means that how fast a protein folds is not something that has been strictly preserved through evolution. In particular, for a type of protein called thioredoxins (THRXs), there is a specific amino acid, proline, at a certain spot (position 76), which slows down the folding process (Leipinšh, 2001). Since the shape that the unfolded protein chain is different from the one that proline encourages, the protein can get stuck before it folds completely. Thioredoxins cannot just swap out this proline for another amino acid because it is crucial for the protein’s active site, which is the part of the protein responsible for its chemical activity, to work properly. Interestingly, when looking at mutations in this region, it turns out that only a change from serine to glycine at position 74 significantly speeds up folding. This might be because glycine is a smaller amino acid without a side chain, allowing the protein more flexibility to twist and turn (Gamiz-Arco, 2019).

### 3.2 Mechanisms of disulfide bond formation and isomerization in bacterial periplasm: the Dsb protein system

**FIGURE 4:**
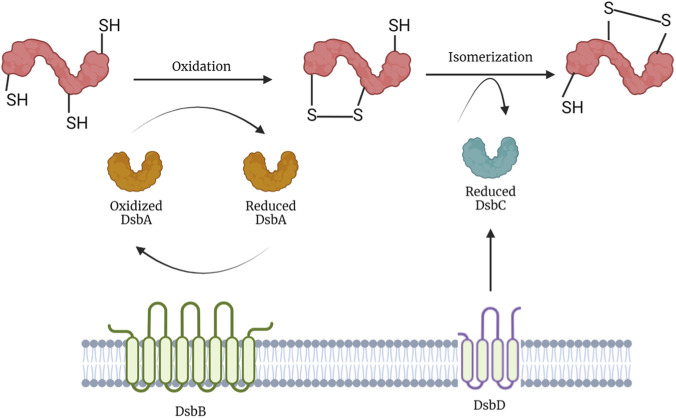
Metabolic pathways of Dsb proteins. The reaction mechanisms are shown in a graphic way. A disulfide bond is introduced by DsbA and is then isomerized by DsbC to the one between the correct cysteine combination.

Disulfide bond formation in bacterial cells is catalyzed by the Dsb protein system. It is composed of numerous proteins located in the periplasm. Seven proteins have been identified and characterized in studies performed in *Escherichia coli* (Luo and Guan, 2012). Five of which are engaged in two complementary, but also competing metabolic pathways: first the oxidation pathway, in which DsbA catalyzes disulfide bond formation and is reoxidized by DsbB, an integral component of the cytoplasmic membrane, as shown in Figure 4 (Villalobos-Alva et al., 2022). The second pathway is that of isomerization/reduction, in which DsbC, DsbD and DsbG participate in the isomerization of non-native disulfide bonds. DsbC and DsbG promote the rearrangement of incorrect disulfide bonds, whereas DsbD transports electrons necessary for catalyzing the isomerization reaction from the cytoplasmic thioredoxin (Domenico et al., 2020). It is now clear that bacterial disulfide oxidoreductases can be monomeric, dimeric, or trimeric (Petit et al., 2022).

Many virulence factors in Gram-negative bacteria are secreted or surface-exposed proteins that rely on disulfide bonds for stabilization (Bocian-Ostrzycka, 2016). As previously discussed, the DsbA/DsbB system is crucial in introducing disulfide bonds across a wide array of virulence factors, making it a key player in mediating bacterial virulence. Genetic studies have demonstrated that bacteria deficient in a functional DsbA are avirulent in animal models of infection. This virulence is attributed to the misfolding and subsequent loss of functionality in proteins that are substrates of DsbA (Adams, 2014).

DsbA acts as the direct donor of disulfides to secreted proteins (Subedi, 2021). Its active site consists of a pair of cysteines present in a Cys30-Pro31-His32-Cys33 motif with a redox potential value of around −120 mV (Inaba and Ito, 2007). This active site can oxidize to form a very reactive disulfide, which is then rapidly transferred to proteins that are in the process of folding. One of the features that contribute to DsbA′s extreme oxidizing power is the very low pKa of its Cys-30 residue (3.5), which makes it a great leaving group in disulfide exchange reactions (Wang, 2012). Another feature is its substrate binding site, which comprises a large hydrophobic groove rather than a well-defined binding pocket (Adams, 2014).

DsbB functions as a cellular apparatus that generates *de novo* protein disulfide bonds by using electrons, which it subsequently transmits to ubiquinone (UQ) or menaquinone (MQ) and ultimately to the terminal oxidases of the respiratory chain (Kadokura and Beckwith, 2002). Embedded within the cytoplasmic membrane *via* its four transmembrane (TM) segments (Inaba and Ito, 2007), DsbB features two periplasmic domains. Each domain contains two crucial cysteines: Cys41-Cys44 in the P1 domain and Cys104-Cys130 in the P2 domain (Wang, 2009). While the Cys104-Cys130 pair actively participates in the redox interaction with the active site of DsbA, the Cys41-Cys11 pair undergoes oxidation by UQ or MQ under aerobic and anaerobic conditions respectively (Inaba and Ito, 2007).

When incorrect disulfides are introduced by DsbA or under conditions of copper oxidative stress, DsbC and DsbG catalyze disulfide isomerization. Both enzymes contain a thioredoxin domain with a CXXC motif, Cys98-Gly99-Tyr100-Cys101 in DsbC and Cys109-Pro110-Tyr111-Cys112 in DsbG (Inaba, 2006). These are linked to an amino-terminal dimerization domain, forming a V-shaped homodimer. These domains fulfill two functions: first, the ability of the catalytic domains to fold independently allows the inside solvent-exposed surface to form a peptide binding cleft that might be important for substrate recognition and its chaperone function. Second, the α-helical linker, which connects the dimerization domain with the catalytic thioredoxin domain, prevents the oxidation of DsbC by the DsbB-DsbA system, maintaining it in a fully reduced state in the periplasm (Jiménez-Roldán et al., 2016).

DsbC and DsbG are maintained in their active reduced form by an interaction with DsbD. It comprises an integral membrane domain of eight transmembrane helices with two periplasmic domains at the N- and C-terminus, respectively. To maintain DsbC and DsbG in a reduced state, a disulfide exchange cascade couples NADPH and cytoplasmic thioredoxin with the cytoplasmic side of the transmembrane domain of DsbD.

DsbA reacts with a newly translocated protein with free thiols, oxidizing it to form a disulfide bond. The reaction can proceed *via* two pathways: a rapid one and a slow one. The rapid one occurs in two steps and involves the covalent mixed disulfide intermediate between DsbA and the substrate. This intermediate reacts very rapidly to either form an oxidized protein or to revert to oxidized DsbA. This thiol-disulfide exchange reaction is extremely rapid, indicating that the catalytic Cys30-Cys33 active site of DsbA is very reactive (Frech et al., 1996). DsbA is then released in the reduced form.

To start a new catalytic oxidation cycle, DsbA is reoxidized by DsbB, a quinone reductase. Electrons then flow from DsbB to UQ and to terminal oxidases, in which UQ selectively oxidizes the Cys41-Cys44 pair to regenerate fully oxidized DsbB. These terminal oxidases transfer the electrons to oxygen in reactions coupled to hydrogen ion transfer and production of water. Under anaerobic conditions, DsbB passes electrons onto menaquinone.

In the slow pathway, the intermolecular disulfide complex undergoes further disulfide rearrangement, in which Cys130 forms a disulfide with Cys41. This intermediate latter with reduced Cys44 (DsbB) and reduced Cys33 (DsbA) is resolved by Cys44 forming a charge transfer complex with UQ as well as a putative adduct complex between them, allowing a nucleophilic attack by Cys41 and thereby the *de novo* formation of the Cys41-Cys44 disulfide (Inaba and Ito, 2007).

Disulfide bonds tend to form preferentially between consecutive cysteines in the polypeptide without regard for the correct pairing of the native conformation. To solve this problem, there is an isomerization system that proofreads and corrects nonnative disulfides. Disulfide bond rearrangement is catalyzed by the thiol-disulfide oxidoreductases DsbC and DsbG, which are maintained in a reduced, catalytically active, state by DsbD. DsbD is reduced by cytoplasmic thioredoxin, which is recycled by thioredoxin reductase (TR) in a NADPH dependent manner. The disulfide exchange occurs directly from the transmembrane domain to the C-terminal domain to the N-terminal domain and ultimately to DsbC or DsbG. Figure 2 summarizes the two pathways followed by Dsb proteins, but these are further described below.

### 3.3 Structural and functional characterization of protein disulfide isomerase: catalytic domains and redox mechanisms

**FIGURE 5:**
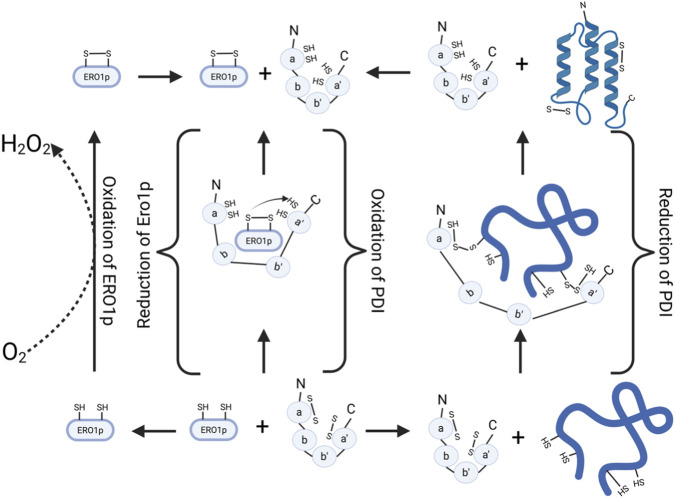
Mechanistic representation of the oxidative folding pathway mediated by PDI and ERO1p. Schematic representation of the redox relay between PDI and ERO1p. Oxidized PDI introduces disulfide bonds into substrate proteins, while ERO1p regenerates oxidized PDI by transferring electrons to molecular oxygen.

PDI functions as a molecular chaperone and a folding enzyme by catalyzing the formation, breakage, and rearrangement of the disulfide bonds in unfolded or misfolded proteins, its mechanism of action is depicted in Figure 5 (Karala et al., 2010).

PDI is a 55-kDa protein that contains a thioredoxin-like domain. Thioredoxins belong to a class of oxidoreductases that contain a dithiol-disulfide active site which participates in redox signaling (Darby and Creighton, 1995). Full length PDI contains 508 amino acid residues which are organized into four domains: a, b, b’, a′ The homologous a and a′ domains contain the active site, CGHC motif (Haugstetter, 2005). The first one is located near the N-terminus and the second one near the C-terminus. The active site cysteines interact with the thiol groups of the substrate, thus mediating the formation and isomerization of protein disulfide bonds. The b and b’ domains assist in the binding of substrates and lack the catalytically active cysteine residues (Horibe, 2004). The domains a′ and b’ are held together by an x-linker region between them. They provide substrate binding sites and undergo an open-closed domain rearrangement depending on the redox state of the a′ domain (Modi et al., 2018). PDI also contains an acidic C-terminus containing a KDEL-ER retrieval sequence (Peixoto, 2018).

The domains that form PDI are arranged in a twisted “U” shape structure, in which the active sites in a and a′ face each other across the long sides of the U. The N-terminal active site cysteines possess a low pKa (4.5) and are solvent exposed making these thiol groups highly reactive. In contrast, the C-terminal cysteines are buried in the interior of the protein and have a high pKa making them unreactive. Domains b and b’ form a rigid base for the U-shaped molecule. The inner surfaces of domains b and b’ form a hydrophobic region which is implicated in substrate recognition. The C-terminus is located at the top of a′ domain facing outwards from the U shape structure (Puig et al., 1994).

The thiol-disulfide balance between a thioredoxin-superfamily protein like PDI and its substrate protein hinges on the relative stability of their dithiol and disulfide forms. PDI’s dithiol state is more stable than its disulfide state, partly due to the low pKa of the cysteine at the N-terminal active site. Each active site’s two cysteines may form an intermolecular disulfide bond, resulting in oxidized PDI, or remain as free thiols in reduced PDI. This state determines whether PDI functions as an oxidoreductase, isomerase, or foldase (Carvalho et al., 2005). Typically, PDIs with higher reduction potentials function as oxidases, while those with lower potentials serve as reductases. The catalysis of disulfide isomerization by PDI thus requires an active site that effectively balances reductase and oxidase capabilities (Robinson et al., 2023).

PDI visits three structurally distinct conformational ensembles, open and closed states that influence its reactivity, consistent with conformational dynamics.

In the oxidase activity PDI inserts disulfides into protein substrates, only one active site is required. It occurs by the pairing of two substrate cysteines into disulfides with concomitant reduction of disulfides of the PDI active site (Irvine, 2014). One cysteine in the substrate is deprotonated to form a reactive thiolate anion, which in turn attacks the disulfide bond between the two cysteines in PDI. This leads to the formation of a transient mixed disulfide between the two proteins that is released by the attack of a thiolate anion derived from the second cysteine in PDI. This reaction is then completed by the protonation of the resulting thiolate anion in PDI (Appenzeller-Herzog and Ellgaard, 2007).

Consequently, the active site cysteines of PDI are reduced by GSH, a reaction that–while also competing with the C-terminal active site cysteines–generates GSSG and contributes to the formation of the glutathione redox buffer in the ER. To proceed efficiently, the pKa of the C-terminal active site cysteines must be low. This differential requirement has been proposed to be resolved by the motion of the side chain of an arginine residue into and out of the active site locale (Karala et al., 2010). The reduction reaction mechanism is the reverse of the oxidation reaction over the catalysis of disulfide bond formation and reduction.

Disulfide bond formation that occurs during protein folding is often error prone, resulting in an incorrect pairing of two cysteines and impeding further folding. PDI catalyzes disulfide isomerization of these bonds into the correct pair of disulfides by breaking and reforming them with different cysteines. The N-terminal active site cysteine initiates the reaction by attacking a non-native disulfide bond and consequently forms a mixed disulfide intermediate between PDI and the substrate. The resulting substrate thiol is free to attack another protein disulfide bond (Darby and Creighton, 1995). The isomerization reaction is driven by energy minimization, where the native disulfide bonds are energetically favored because of non-covalent bonds like hydrogen bonds or ionic interactions. These are dictated by the protein’s primary structure, making the native conformation the most thermodynamically stable. This also allows the reaction to occur faster than the C-terminal active site reaction of the same non-native disulfide bond, which would result in reduction. Once the catalysis reaction is over, PDI is regenerated into its original reduced state (Neves et al., 2017).

To act as an efficient isomerase, the N-terminal active site cysteine of PDI must have a low pKa. While isomerization can occur *via* cycles of oxidation and reduction, direct reaction is favored by a high pKa value of the C-terminal active site cysteine. The lifetime of the mixed disulfide state depends on both the N- and C-terminal active sites pKa and the affinity of the substrate binding sites of PDI (Karala et al., 2010).

PDI can distinguish between partially folded, unfolded, and properly folded protein substrates (Forester, 2009). It has a high affinity towards misfolded proteins rather than native proteins. It binds misfolded proteins through hydrophobic interaction. This property and its conformational flexibility make PDI a highly effective chaperone. In the ER, it binds misfolded proteins and activates the unfolded protein response (UPR). The main function of UPR is to reduce the concentration of unfolded proteins by decreasing protein biosynthesis. Another of its functions is to induce the activity of PDI and other chaperones to increase protein folding capacity in the ER (Puig et al., 1994). This protective mechanism can become dangerous since a prolonged activation of UPR can lead to apoptosis of the cell (Kersteen et al., 2005).

There are many other functions in which PDI plays a part in. For example, it facilitates the degradation of misfolded proteins *via* the ER associated degradation (ERAD) pathway, which occurs by translocation of proteins from the ER to the cytoplasm. The ubiquitin proteasome system is responsible for the degradation process (Honjo et al., 2010). PDI also helps in protein quality control by retaining unassembled proteins until the correct native structure is formed it as well can bind transiently or permanently to other proteins to stabilize its native conformation, such as the ß subunit of collagen prolyl 4-hydroxylase (Honjo et al., 2010). Depending on its initial concentration, PDI can show either chaperone or anti-chaperone activity. In experiments where the initial concentration is high, basically all the protein substrates were correctly folded. On the other hand, when the initial concentration is low, it can promote intermolecular disulfide cross linking among protein substrates. This leads to the formation of aggregates in the cell (Sideraki and Gilbert, 2000).

### 3.4 Experimental approaches for assessing the oxidoreductase, isomerase, and chaperone activities of PDI

There are several ways to measure PDI activity. Some assays are more specific to thiol reduction or oxidation activity while others focus on the measurement of the isomerase activity. These assays have three main purposes; The first one is to identify substrate intermediates to understand the mechanism of reaction. This requires analysis and detection by mass spectroscopy. The second one is to screen PDI substrates or inhibitors, usually by high-throughput system (HTPS) platforms. Finally, the third main objective is to understand PDI function in a physiological context by comparison of its activities in different experimental conditions in biological samples.

Proteins such as bovine pancreatic trypsin inhibitor (BPTI) and ribonuclease A (RNAse) are mainly used as substrates in studies of PDI-mediated protein folding, while insulin is used for HTPS automation. However, PDI assays in biological samples are considered a huge challenge Some substrates commonly used for these assays include insulin, fluorescent glutathione (GSSG) (Inaba and Ito, 2007), and lysozyme. These substrates are also already used in cell homogenates Nevertheless, the interpretation is still difficult due to the presence–or lack thereof–of other reductants in the assay, this is also a consequence of the difficulty to imitate the exact physiological conditions in which the PDI functions on vivo.

There are other peptide fragments that have been developed to measure PDI activities *in vitro*. These are linked to fluorescent moieties that allow high sensitivity and direct measurement of fluorescence. An example is a peptide based on tachyplesin I (TI); a 17-residue antimicrobial peptide that crosses the membranes. Scrambled TI, which has 4 cysteine residues, is isomerized by PDI to its native conformation allowing fluorescence resonance energy transference (FRET) (Kersteen et al., 2005).

Assays to measure the isomerase activity of PDI are based on the regain of function of an inactive protein substrate containing scrambled disulfide bonds. The most used enzyme as a substrate is scrambled RNAse. For its preparation, it is reduced in denaturing conditions and then allowed to oxidize at room temperature for it to acquire random (non-native) disulfide bonds. After being incubated with PDI, substrate activity recovery is measured by the hydrolysis of an RNAse substrate, such as cyclic cytidine monophosphate (cCMP), which provides high increases in absorbance (Hawkins and Freedman, 1991). The assay buffer generally contains the redox pair GSH/GSSG to imitate physiological conditions. One disadvantage of this method is the lack of reproducibility of the results due to the heterogeneity of the substrate among preparations. For this reason, it is highly desired to obtain all experimental results from the same SRNAse batch.

Several fully reduced proteins can be used as substrates for PDI mediated-oxidative refolding. RNAse and BPTI, which have 3 disulfides each, are considered the best model substrates for an oxidative folding assay (Karala et al., 2010). In both cases, the intermediates formed during the reaction are characterized by mass spectroscopy after thiol alkylation (Irvine, 2014). This is the most effective method to estimate rate constants of PDI-dependent reactions during different steps of the folding process. Another frequently used substrate is lysozyme, which contains four disulfides. Translation to biological samples has the same difficulties as those described in the isomerase activity.

In these assays, the opposite happens. This means that PDI will reduce disulfide bonds present in the oxidized substrate. On the other hand, insulin is the most used due to its low cost and technical simplicity. The reduction of this substrate promotes the aggregation of an insulin B chain and is followed by an increase in turbidity. It is also the method used for screening PDI inhibitors and can be optimized for HTPS or coupled to fluorogenic dye (Tan, 2005). To improve assay quantification, it is possible to couple insulin reduction with NADPH consumption by coupling with GSSG or thioredoxin reductase. In this case, one enzyme unit is defined as the amount of PDI that catalyzes the formation of GSSG per minute, allowing the quantification of kinetic parameters, nevertheless, this assay must be optimized in a way that insulin aggregation does not interfere with NADPH absorbance (Xu et al., 2013).

Assays for chaperone-like activity of PDI are based on the catalysis of the self-refolding process of completely denatured substrates. The first method proposed to distinguish chaperone from isomerase activity is the use of D-glyceraldehyde-3-phosphate dehydrogenase as a substrate. Another substrate that can be used is lactate dehydrogenase (Xu et al., 2013). The denatured protein is diluted in GSH/GSSG buffer, in the presence of excess PDI. Changes in aggregation are followed by light scattering or turbidity. Since changes in aggregation may not correlate with efficient substrate regain of function, the reactivation of the substrate is also measured.

### 3.5 Impact of active site mutations on PDI functional activity and substrate interactions

Through the modification of the active site from CGHC to CGHA, the stabilization of the PDI-substrate intermediate was possible and allowed for PDI substrates identification because the process was slower through the mutation (Stopa et al., 2017). The limitation towards this pro-longed intermediate, is the inability to identify substrates oxidized by PDI. Since it only identifies reduced substances, PDI can act as an oxidoreductase and as an isomerase depending on the relative stability of the dithiol and disulfide states. Through the stabilization of the PDI-substrate intermediate it is possible to identify the substrate’s affinity for oxidation or reduction by PDI.

PDI has 2 active sites with 2 reactive cysteine residues each; to enhance or inhibit certain PDI reaction mechanisms, several mutations have been studied where the cysteine residues are replaced with serine residues under different combinations of C-terminal and N-terminal active sites (Gao, 1997).

When the second cysteine in the PDI active site is mutated to Ser the isomerase activity decreases 7-8-fold and covalent intermediates with substrates accumulate (McCarthy, 2000). Hence the C-terminal active site provides an escape mechanism that reduces the number of substrates bound for PDI through the reduction and reoxidation cycles that prevent the saturation of PDI with slow isomerizing substrates.

It was found through modification of the two cysteines in the C-terminal active site regions and alkylation of the sulfhydryl groups with iodoacetate that peptide binding is independent of the a′ domain. Through mutations like this, the b domain was identified as the main region of substrate binding. Since then, it has been utilized for quantitative determination of PDI activity and specificity with 10-fold differences about affinity (Noiva et al., 1993). In contrast to mutations on the N-terminal active site, there is no impact on the capacity to perform redox reactions.

When the C-terminal cysteines are replaced with alanine residues the effect contrasts with the loss of isomerization since the reduction and oxidation of substrates diminishes maintaining the isomerase activity of the wild-type PDI (Kersteen et al., 2005). This means that the C-terminal cysteine residues are insignificant towards the isomerization of disulfide bonds. A similar effect is obtained through modification of Arg120 and Arg 461 which are responsible for modulating the pka of the C-terminal active site cysteine and the reactivity of the N-terminal active site. Due to the fact that the ability to catalyze disulfide bond formation and reduction diminishes as the catalysis for isomerization reaction increases, there is a more stable formation of mixed complexes (Karala et al., 2010).

A mutant with an inactivated C-terminal active site exhibits nearly wild-type enzymatic activity, with a catalytic rate kcat of 0.73 min^−1^ and a Michaelis constant Km of 29 μM, compared to the wild types kcat of 0.76 min^−1^ and Km of 6.9 µM. Conversely, a mutant with an inactivated N-terminal active site shows a reduced catalytic rate of 0.24 min^−1^ but maintains a nearly wild-type Km of 7.1 µM. These observations suggest that the C-terminal region plays a more significant role in substrate binding under steady state conditions, while the N-terminal region is more crucial for catalysis (Kadowaki and Nishitoh, 2019).

As groundbreaking as PDI can be towards the treatment of protein folding diseases, PDI can act as an anti-chaperone through misfolding and aggregation of its substrate under specific conditions. The anti-chaperone behavior occurs at low concentrations of PDI (1–5 µM) that react with denatured lysozyme at a concentration range of 10–50 µM. It was found that PDI promoted aggregation occurs at a lysozyme-PDI precipitation ratio of 10:1 even though the presence of dithiothreitol (10 mM) precludes disulfide formation (Kersteen et al., 2005). Hence, to revert the anti-chaperone behavior of PDI there must be an increase in the concentration of PDI with respect to the concentration of denatured lysozyme. Similarly, when both the N-terminal and C-terminal active sites cysteines are changed to serines, all chaperone activity is lost at all substrates and PDI concentrations because the dithiol/disulfide sites are essential for chaperone behavior, but not for anti-chaperone activity (Puig et al., 1994).

PDI can also become dysfunctional through post-translational modifications that place oxidative stress on the redox active cysteine residues. The biological oxidant peroxynitrite oxidizes the cysteine residues into the sulfenic acids that react with thiols forming disulfides. Oxidative stress causes the inactivation of PDI *via* oxidation, nitration, and covalent oligomerization.

To regulate PDI anti chaperone activity, cysteine containing peptides have been found to have 4-8-fold better inhibitors than other peptides of same length. For PDI inhibitor peptide length is the main factor over composition, hydrophobicity, and charge. However, chaperone activity can be blocked just like nitric oxide inhibits PDI by inducing S-nitrosylation, allowing protein misfolding to occur in patients with Alzheimer’s disease.

### 3.6 Conserved structures in PDI: phylogenic and primary structure analysis

The human PDI family includes multiple paralogs with thioredoxin-like domains, including ERp57, ERdj5, ERp44, and ERp72, each with distinct redox or chaperone roles. For example, cysteine-rich proteins with epidermal growth factors (EGF)-like domains contain CXXC motifs and can isomerize disulfide bonds but share little similarity with PDIs that have thioredoxin-like domains. The crystal structure of human PDIA1 reveals four thioredoxin-fold domains arranged in an abb′xa’ configuration.

Based on the amino acid sequence of the 29 selected proteins, several distinct clades can be identified. The first clade includes PDIA1 and PDIA2 from *Caenorhabditis elegans*, with bootstrap value of 81, indicating a moderately reliable evolutionary relationship despite having less than 70% sequence similarity. The second clade consists of PDIA2 proteins, which show a highly reliable evolutionary relationship with PDIA1 (bootstrap value of 99). In fact, the identity matrix reveals more than 50% sequence similarity between these two clades, which is not observed among the remaining clades. The third clade comprises PDIA5 proteins, though their evolutionary relationship with PDIA1 and PDIA2 is weakly supported, with a bootstrap value of 25.

The fourth clade consists of yeast PDIs, which may be related to more ancestral forms of PDI. The fifth clade contains only PDI1 from *Dictyostelium discoideum*. The sixth clade includes PDIA6 proteins, where *Drosophila melanogaster* (fruit fly) exhibits less than 70% similarity, likely due to the evolutionary distance between this organism and mammals. The seventh clade consists of PDI from *Hordeum vulgare* and PDI11 from *Arabidopsis thaliana*, while the eighth and ninth clades correspond to PDIA1 and PDIA3 from mammals, respectively.

Despite its utility, the phylogenetic tree has limitations, some bootstrap values are very low, making it difficult to determine the evolutionary relationship between certain clades with confidence. Future studies could refine sequence selection criteria to construct a more informative phylogenetic tree that provides deeper insights into the evolutionary history of PDIs.

Eleven proteins from the cladogram were selected for further analysis, ensuring representation from different organisms and choosing one member from each of the 11 groups identified by CD-HIT. Table 1 summarizes key characteristics of the selected PDI proteins, including their UniProt ID, short name, species of origin, number of amino acid residues, molecular mass (in Daltons), disulfide bond positions, and domain organization.

**TABLE 1:** Generalities of the 11 selected PDIs.

UniProt code	Name	Organism	Length (aa)	Mass (Da)	CXXC Motif	Domains
P17967	PDI	*Saccharomyces cerevisiae*	522	58,227	61–64; 406–409	abb’xa′-HDEL
Q86IA3	PDI1	*Dictyostelium discoideum*	363	39,905	51–54; 172–175	aa′b-FKSK
Q9XI01	PDI11	*Arabidopsis thaliana*	501	55,602	59–62; 404–407	abb’xa′-KDEL
Q17770	PDI2	*Caenorhabditis elegans*	493	55,152	52–55; 393–396	abb’xa′-HDEL
P05307	PDIA1	*Bos taurus*	510	57,266	55–58; 399–402	abb’xa′-KDEL
D3Z6P0	PDIA2	*Mus musculus*	527	58,316	74–77; 421–424	abb’xa′-KEEL
Q4VIT4	PDIA3	*Chlorocebus aethiops*	505	56,779	57–60; 406–409	abb’xa′-QEDL
P38659	PDIA4	*Rattus norvegicus*	643	72,720	89–92; 204–207; 553–556	aa′bb’xa′’-KEEL
Q14554	PDIA5	*Homo sapiens*	519	59,594	182–185; 305–308; 426–429	baa′a′’-KEEL
P38660	PDIA6	*Mesocricetus auratus*	439	48,161	55–58; 190–193	aa′b-KDEL
Q9V438	PDIA6H	*Drosophila melanogaster*	433	46,752	55–58; 186–189	aa′b-KDEL

Table 1 shows that while most PDI proteins are approximately 500 amino acids long, PDIA4 in rats is significantly longer, whereas PDI2 in amoebas is much shorter. Additionally, some PDI proteins have more than two disulfide bonds, although it is unclear whether these extra bonds also play a role in oxidoreduction or disulfide isomerization.

The Clustal Omega alignment revealed 25 highly conserved amino acids, 29 moderately conserved, and 16 weakly conserved.

GBlocks identified five conserved blocks within the PDI sequences, which were visualized using Jalview. Figure 7 displays the amino acids categorized by color according to their physicochemical properties.

The amino acids adjacent to the CXXC motif not only share similar physicochemical properties but are also nearly identical across all analyzed structures. Additionally, the yellow bars in Figure 7 highlight three conserved contiguous amino acid segments, two of which are located near the CXXC motif.

A total of 99 amino acids contribute to the consensus structure. However, not all 29 conserved amino acids identified by Clustal Omega are included, as GBlocks excluded some that were not considered relevant for conserved region analysis.

### 3.7 Conserved structures in PDI: secondary structure analysis

The experimentally determined structure of yeast PDI and the AlphaFold-predicted structures of the remaining PDI proteins were uploaded in PDB format to Dali, using the “pairwise structure comparison” tool.

**FIGURE 6:**
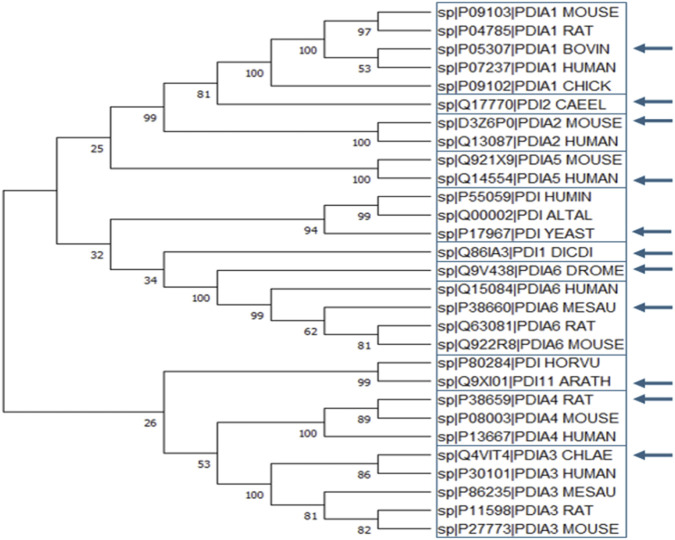
Phylogenetic tree of 29 PDI proteins from various organisms. Boxes indicate proteins with at least 70% sequence similarity, while arrows mark the selected PDI proteins used in subsequent analyses. Bootstrap values are displayed near each node. The organism abbreviations are as follows: *H. sapiens* (HUMAN), *S. cerevisiae* (YEAST), *Mus musculus* (MOUSE), *D. melanogaster* (DROME), *Mesocricetus auratus* (MESAU), *Rattus norvegicus* (RAT), *Arabidopsis thaliana* (ARATH), *Caenorhabditis elegans* (CAEEL), *Gallus gallus* (CHICK), *Bos taurus* (BOVIN), Hordeum vulgare (HORVU), Humicola insolens (HUMIN), *Dictyostelium discoideum* (DICDI), Chlorocebus aethiops (CHLAE), and *Alternaria alternata* (ALTAL).

As a result, a consensus secondary structure was obtained for PDI proteins and a separate consensus for their prokaryotic homologs: the Dsb proteins. Figure 6 illustrates the comparison between structures.

Overall, the secondary structures of PDI proteins are highly conserved, with major differences observed in proteins that contain more or fewer domains. Since these proteins are entirely or partially composed of thioredoxin-like domains, the number and position of alpha-helices and beta-strands remain largely unchanged despite differences among PDI family members and organisms.

Figure 6 shows the consensus secondary structure of PDI proteins, as determined by Dali. To confirm the conservation of an α-helix or β-strand, it needed to be present in at least 6 of the 11 aligned PDI proteins. The same approach was applied to Dsb proteins, but in this case, a structure was considered conserved if it appeared in at least 5 of the 9 aligned sequences.

A sequence alignment in UniProt revealed that β1 in Dsb proteins corresponds to β16 in PDI proteins, while β2 in Dsb is homologous to β17 in PDI. Additionally, α12 in PDI is absent in prokaryotic proteins. Another difference is that in Dsb proteins, α2 and α3 are small and located adjacent to each other, whereas in PDI, they form a single larger helix (α13). These secondary structures contain the CXXC motif. Finally, β3 in Dsb aligns with β18 in PDI, but it appears that α4 in prokaryotes evolved into β19 in eukaryotes.

Table 2 is provided to facilitate the identification of the consensus structure for each conserved block.,. The positions correspond to the Clustal Omega alignment, while the consensus structure was determined using Jalview. In the table, the “+” symbol indicates amino acids where conservation could not be clearly determined across aligned structures and therefore, were not included in the consensus.

**TABLE 2:** Consensus of each conserved block based on physicochemical properties in the primary structure of PDI and its location according to the secondary structure consensus.

Block number	Alignment position	Primary structure consensus	Location in secondary structure
1	315–339	VEFYAPWCGHCKKLAPEYEKAATAL	β2 y α2
2	362–380	LA + Q + GVSGYPTLKIFRNG	α3 y β4
3	388–397	YNGPRTA + GIV	α4
4	676–688	KVLVGKNFDE + V	β16 y α12
5	690–721	D + KKNVLVEFYAPWCGHCK + LAP + WD + LAEKY	β17 y α13

### 3.8 Conserved structures in PDI: tertiary structure analysis

An “all-against-all” comparison was made in Dali, and using the three-dimensional structure data, a similarity matrix was generated with a scale from 0 to 50, the higher the value, the greater the similarity between the proteins.

The tertiary structures of selected PDI proteins were analyzed using a combination of experimentally determined and computational models. The crystal structure of human PDIA1 (PDB: 4EL1) reveals a modular domain arrangement comprising four thioredoxin-like domains (a, b, b′, a′) and a flexible x-linker between b′ and a′. This arrangement forms a twisted “U”-shaped conformation, which facilitates substrate binding and domain motion during catalysis.

Structures of bacterial homologs such as DsbC (PDB: 1EEJ, 1T3B, 4I5Q) and DsbG (PDB: 1V57, 3TGD, 4IHU) were also included to highlight conserved folding motifs and active-site arrangements. DsbC forms a homodimeric V-shaped structure, with each monomer containing a single thioredoxin fold; the dimer interface plays a critical role in substrate specificity and isomerization activity.

Structural alignments using Dali revealed a high degree of conservation in the thioredoxin core fold across the PDI family, despite differences in the number and position of redox-active domains. Conserved catalytic motifs (CXXC) were mapped to solvent-exposed loops within α-helix/β-sheet frameworks, suggesting a shared mechanism of disulfide exchange.

**FIGURE 7:**
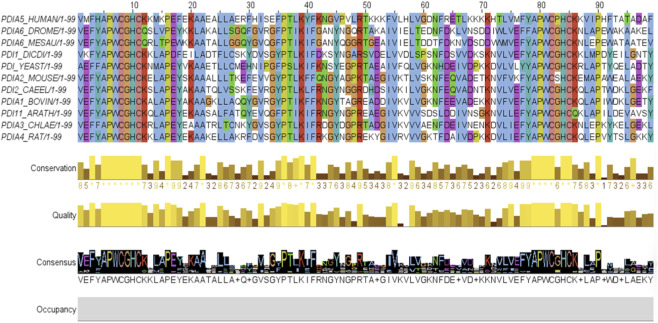
Alignment of conserved blocks identified by GBlocks. Hydrophobic amino acids are shown in blue, positively charged hydrophobic amino acids in red, negatively charged hydrophobic amino acids in magenta, polar hydrophobic amino acids in green, cysteines in pink, glycine in orange, prolines in yellow, aromatic residues in cyan, and non-conserved amino acids in white. The conservation proportion is represented by yellow bars, and the consensus structure is displayed at the bottom.

**FIGURE 8:**
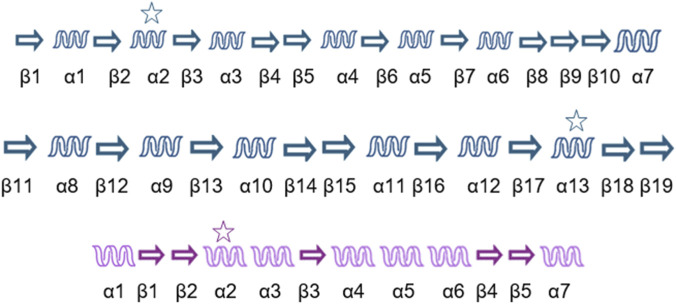
Conserved secondary structures in PDI and Dsb proteins. The PDI secondary structure consensus is shown in blue (top), while the Dsb consensus is in purple (bottom). Stars indicate the helices containing the CXXC motif.

**FIGURE 9:**
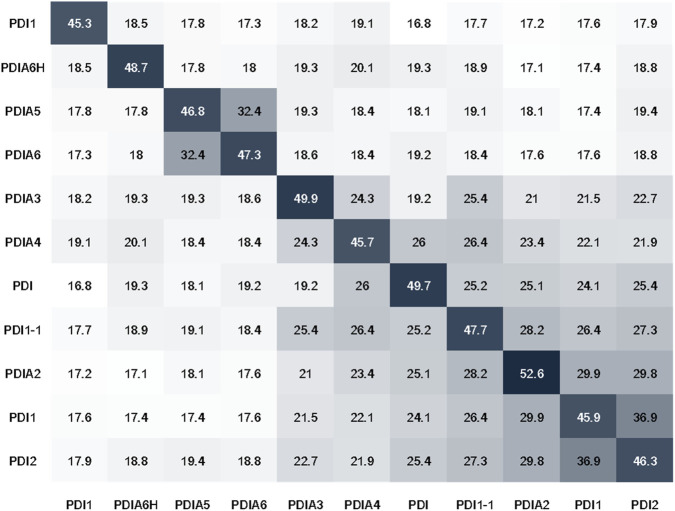
Structural similarity matrix of PDI proteins. The labels are the same as those used in Table 1. PDB codes: PDIA1 (human) – *4EL1*, PDIA3/ERp57 – *3F8U*, PDIA4/ERp72 – *3L1N*.

**FIGURE 10:**
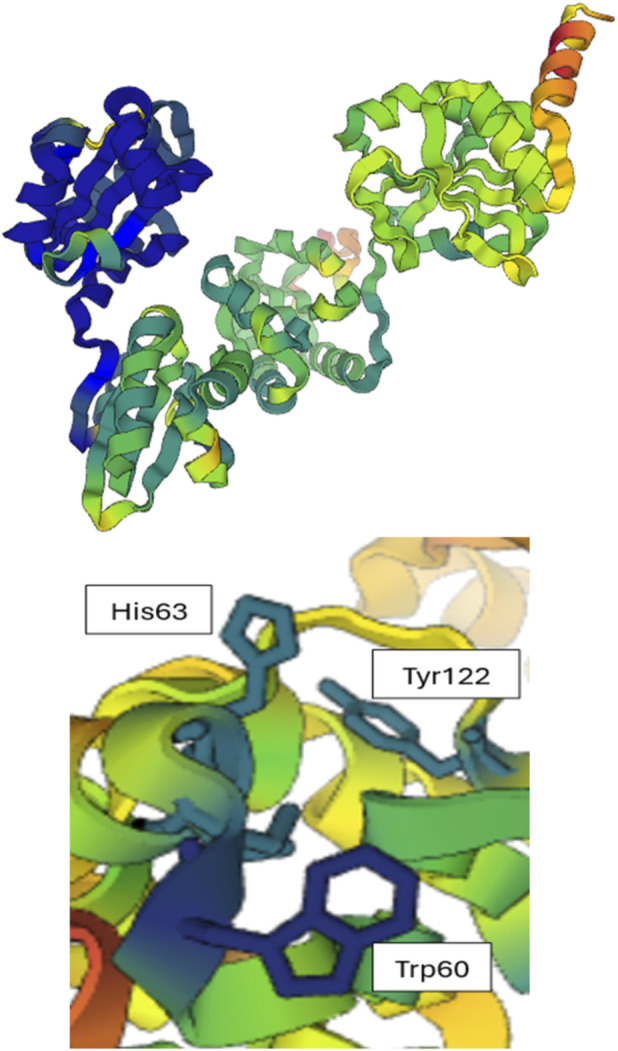
Conservation of sequence and tertiary structure in the selected PDI proteins. On the bottom, a close-up of the PDI active site highlights five conserved amino acids: Cys61, His63, Cys64 (part of the CXXC motif), Trp60, and Tyr122, all numbered based on the yeast PDI sequence. On the left, the conservation of tertiary structures is shown, where blue and green regions represent highly conserved areas, while yellow and red regions indicate low conservation. PDB codes: *Escherichia coli* DsbC – *1EEJ*, *Haemophilus influenzae* – *1T3B*, *Salmonella enterica* – *4I5Q*.

Figures 7–10 depict superpositions of PDIA1 with DsbC and DsbG, highlighting conserved tertiary folds and spatial arrangements of catalytic residues. These overlays emphasize that while bacterial and eukaryotic proteins share a common fold, variations in domain architecture and flexibility confer specialized functions in different cellular environments.

The functional diversity of the PDI family arises from variations in their domain architecture, which directly influence substrate specificity, redox activity, and protein–protein interactions. Canonical PDI proteins such as PDIA1, feature four thioredoxin-like domains (a, b, b′, a′) arranged in an abb′xa’ configuration where the a and a′ domains contain redox-active CXXC motifs responsible for disulfide exchange. The b and b’ domains contribute to substrate binding and structural flexibility. In contrast, ERp57 lacks the x-linker and works in concert with the calnexin/calreticulin cycle to fold glycoproteins, whereas ERp44 contains a single active domain and primarily acts as a redox-sensitive chaperone. ERdj5 includes a J-domain that recruits the ATPase BiP, linking redox isomerization to the ER stress response. The presence or absence of catalytic domains, ER-retention signals, and oligomerization interfaces shapes each protein’s cellular function. Importantly, these architectural features are also tied to disease pathology: for example, PDIA1 dysfunction through S-nitrosylation has been implicated in Alzheimer’s disease, and ERp57 overexpression is frequently observed in cancers, where it may contribute to enhanced survival and redox adaptation of tumor cells. Understanding how domain architecture modulates PDI family activity is therefore essential not only for grasping redox biology but also for identifying therapeutic targets in diseases driven by protein misfolding and ER stress.

## 4 Discussion

### 4.1 Systematic review: knowledge gaps and strategic priorities

Our PRISMA-guided survey of 96 primary studies provides the first consolidated, cross-disciplinary portrait of the PDI/Dsb field. Three main insights emerged: (i) Across organisms, experimental work is strongly skewed toward a handful of canonical enzymes—human PDIA1, yeast ScPDI, *Escherichia coli* DsbA/B/C, and ERp57—while >70% of annotated paralogues have never been biochemically characterized. (ii) Only 11 thioredoxin-fold proteins (≈15% of family members with a CXXC motif) possess any high-resolution 3-D structure, and in 10 of those cases the structure is restricted to a single domain; whole-protein architectures, redox-dependent conformational states and transient substrate complexes remain largely inferential. (iii) Disease connections are reported in scattered, single-enzyme studies (e.g., PDIA1 in Alzheimer’s, ERp57 in cancer, ERp44 in thrombosis), but there is almost no comparative work linking domain architecture to pathology across the family. Performing the systematic review was therefore essential for mapping where evidence is dense *versus* absent, exposing critical blind spots, particularly the paucity of kinetic data for non-canonical PDIs, the lack of in-cellulo validation of AlphaFold models and the inconsistent nomenclature that hampers meta-analysis. By integrating these literature gaps with the bioinformatic analysis, the study provides an epistemic framework that prioritizes: (a) full-length structural determination of under-studied isoforms, (b) unified *in-vitro* assay conditions for catalytic efficiency, and (c) mechanistic studies that relate domain composition to disease phenotypes.

### 4.2 Structural and functional conservation in PDI proteins

The present bioinformatic analysis was designed to address three critical knowledge gaps that cannot be resolved by the current experimental record alone. First, structural scarcity: 10 of the 11 representative PDIs we selected have no crystallographic or cryo-EM models in the PDB. By generating AlphaFold-2 structures for these orphan homologues, we supply the only available 3-D templates for roughly one-third of the reviewed PDI repertoire. Second, phylogenetic breadth: the final panel deliberately spans fungi, plants, metazoans, and protozoa, enabling an evolutionary comparison that single-species studies cannot achieve. Third, functional blindness: for most of these orthologues even basic kinetic or binding data are missing; our structure-based landscape therefore delivers the first testable hypotheses on catalytic geometry, redox potential, and substrate access channels.

Integrating the new models with the solved structures of PDIA1, DsbC, and DsbG uncovered a core thioredoxin fold that is strictly conserved despite radical differences in domain copy number, redox motifs, and cellular niche. Mapping these alignments onto phylogeny allowed us to pinpoint lineage-specific insertions and deletions that likely tune isomerase *versus* oxidase bias, and to flag previously unnoticed allosteric helices that could regulate activity. Taken altogether, the atlas we provide (i) closes a major structural vacuum, (ii) supplies an evolution-aware framework for rational mutagenesis and inhibitor design, and (iii) opens an epistemic horizon for biochemical work, prioritizing residues, loops, and inter-domain contacts whose roles can now be interrogated experimentally.

Our comprehensive bioinformatic analysis identified significant conserved structural elements across the Protein Disulfide Isomerase (PDI) family, providing key insights into their biochemical mechanisms and physiological roles. Through primary sequence alignment performed using Clustal Omega, we pinpointed 25 highly conserved amino acids, underscoring their direct contributions to enzymatic stability and catalytic functionality. These conserved residues predominantly cluster around the catalytic CXXC motifs, reinforcing their importance in catalysis, redox cycling, and substrate interactions.

The structural superposition conducted using the Dali server identified key conserved amino acids and structural features, which are shown in Figure 8.

Unsurprisingly, a domain is the most conserved, as it contains the CXXC motif, which is essential for oxidoreductase and isomerase activity. Additionally, the b and b' domains are also highly conserved within this protein family. This suggests that, despite differences in amino acid composition among PDI members, the three-dimensional structure of these domains remain preserved due to their physicochemical properties.

In contrast, the a' domain is far less conserved than the a domain. One possible explanation is the structural flexibility of PDIs, as their three-dimensional conformation depends on various factors, such as phosphorylation and redox state, which cannot be fully accounted for in AlphaFold structural predictions.

To further investigate conservation, the segments identified by GBlocks were mapped. Three of these conserved segments were found in the a domain, while two were in the a' domain. Figure 9 presents the ChimeraX visualization of both domains.

The comparison between the a and a′ domains reveal intriguing differences. Such as, in the a domain, three conserved fragments surround the active site, forming loops that contain key amino acids. These include a tryptophan preceding the CXXC motif, an arginine near position 120 that lowers the pKa of the C-terminal cysteine in the motif, and a proline near position 76 that slows the folding process of PDI. Each of these residues is in a different loop adjacent to the active site.

In contrast, in the a' domain, only one of the three loops surrounding the active site is conserved. Instead, a small alpha-helix at the top of the domain (see Figure 9) appears to be highly conserved. This region is too distant from the active site to play a direct catalytic role, and due to its position, it is unlikely to be involved in substrate binding. However, it may participate in the allosteric regulation of PDI, potentially interacting with an effector molecule.

**FIGURE 11:**
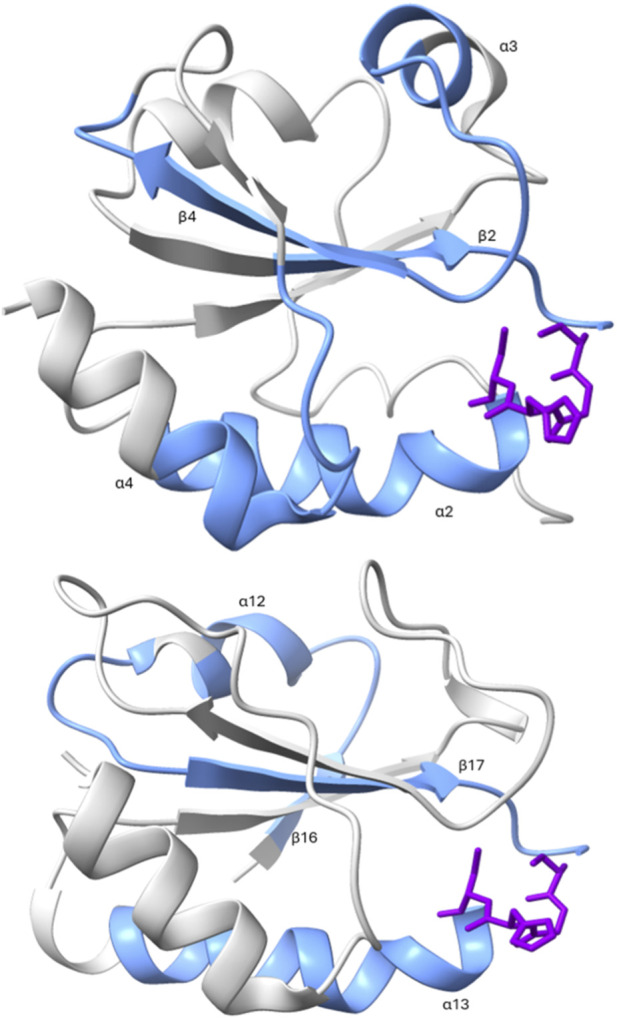
Visualization of conserved blocks at the tertiary structure level. Conserved regions in the catalytic domains of PDI proteins are shown in blue. On the left, the a domain is displayed, while on the right, the a' domain is shown. The CXXC motif is highlighted in purple. PDB codes: *Escherichia coli* DsbG – *1V57*, *Helicobacter pylori* – *3TGD*, *Mycobacterium tuberculosis* – *4IHU*.

These findings suggest that for engineering a PDI with enhanced isomerase activity, special attention should be given to the three loops surrounding the active site in the a domain, considering both their amino acid composition and their interactions with the catalytic motif (Figures 10, 11).

**FIGURE 12:**
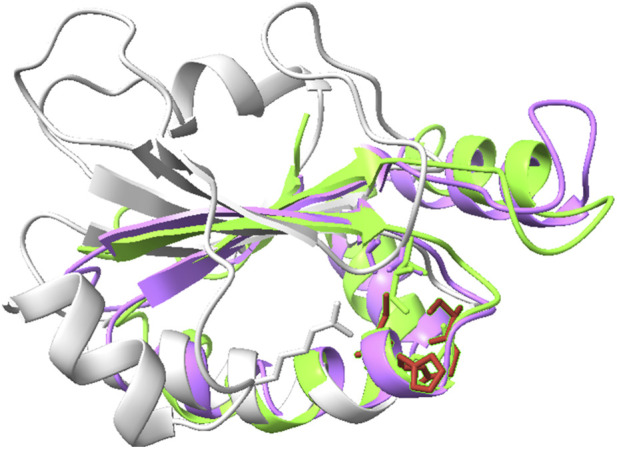
Comparison of human PDIA1 (gray), DsbC (purple), and DsbG (green) from *Escherichia coli*. The CXXC motif of PDI is highlighted in red. PDB codes: PDIA1 – *4EL1*, DsbC – *1EEJ*, DsbG – *1V57*.

Additionally, the three-dimensional structures of PDIA1, DsbC, and DsbG were superimposed using ChimeraX (see Figure 12). This comparison clearly highlights secondary structures that are similarly arranged in space, as well as those that differ. Notably, two of the highly conserved loops in PDI are absent in Dsb proteins.

Finally, Table 3 presents the Km values of different PDI proteins, along with the substrate used in the assay and the specific function of PDI that was measured. Additionally, an image of the active site surface of each protein is included to identify potential patterns between the hydrophobic surface near the active site and substrate affinity.

**TABLE 3:** Surface of PDI Active Site. K_m_ values have been determined.

Protein	PDI from bovine liver	FhPDI	PDIA1	PDIA1 (with loss of 2 Cys in a′ domain)
Activity	Isomerase	Oxidase	Isomerase	Isomerase
Substrate	RNAse scrambled	Lysozyme reduced	RNase denaturized	RNase A
Specific activity (µmol/min/mg)	7	Not reported	Not reported	Not reported
Km (µM)	20.0	7.0	27.8	30.0
Organism	*Bos taurus*	*Entamoeba histolytica*	*Homo sapiens*	*Homo sapiens*
Active site	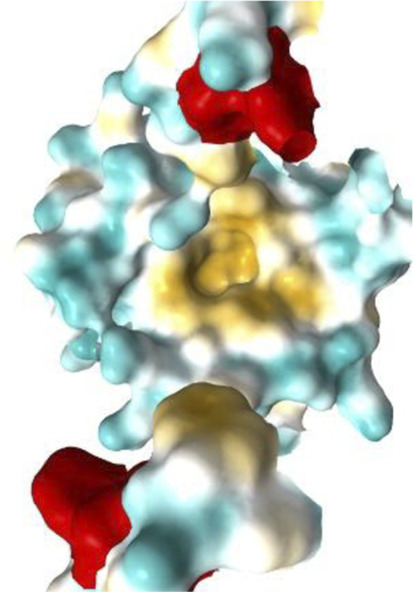	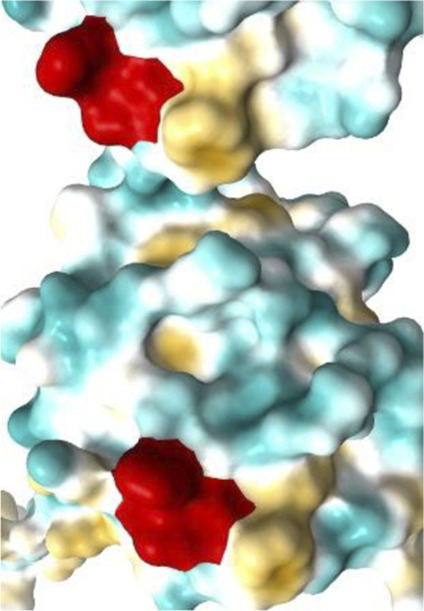	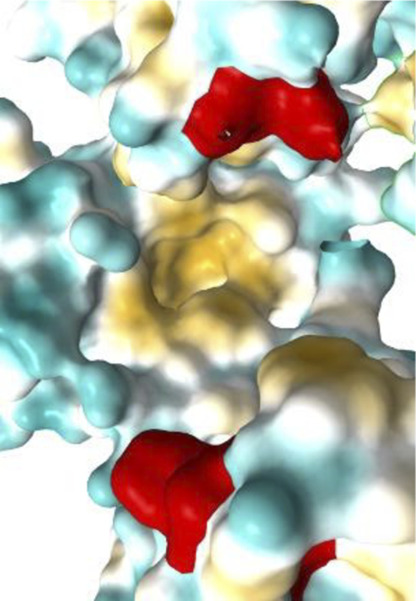	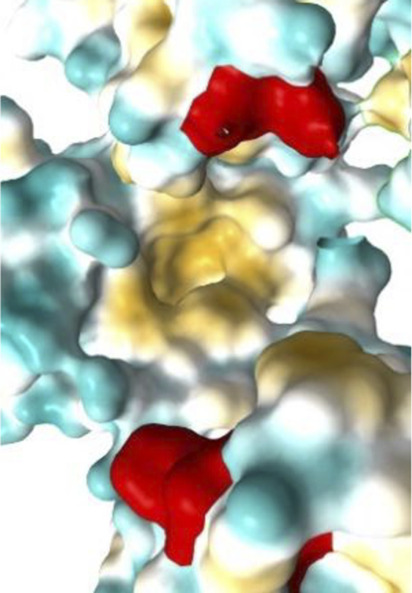

Protein	PDIA1 (with loss of 2 Cys, one in a domain and one in a′ domain)	Thioredoxin	NCgl2478	FhPDI
Activity	Isomerase	Isomerase	Reductase	Reductase
Substrate	RNase A	RNase denaturized	Hydroxymethyl disulfide and 2-hydroxymethyl disulfide	Insulin
Specific activity (µmol/min/mg)	Not reported	Not reported	Not reported	Not reported
Km (µM)	50.0	66.5	510.0	2026.0
Organism	*Homo sapiens*	*Homo sapiens*	*Corynebacterium glutamicum*	*Entamoeba histolytica*
Active site	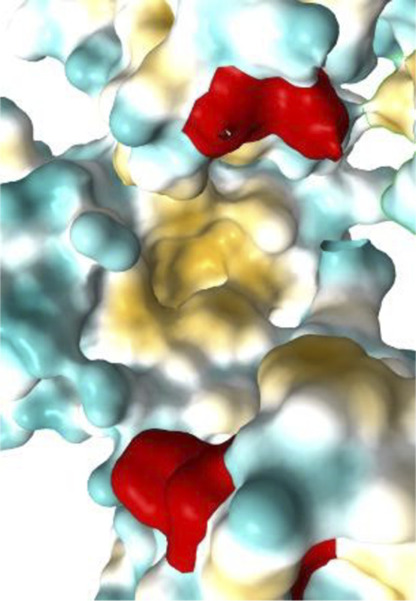	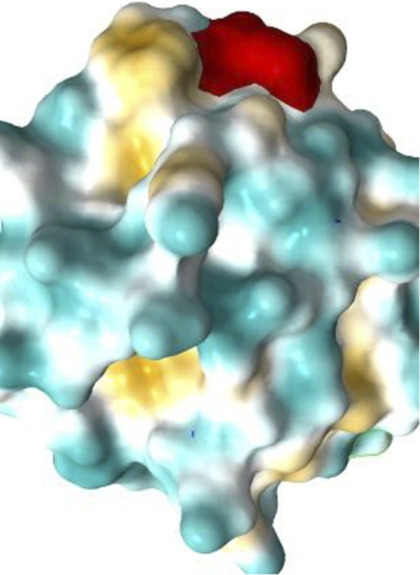	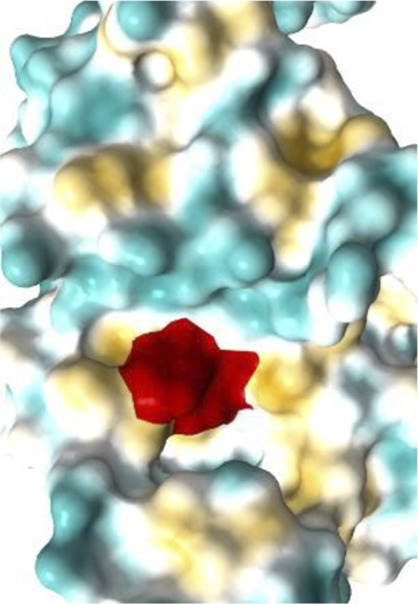	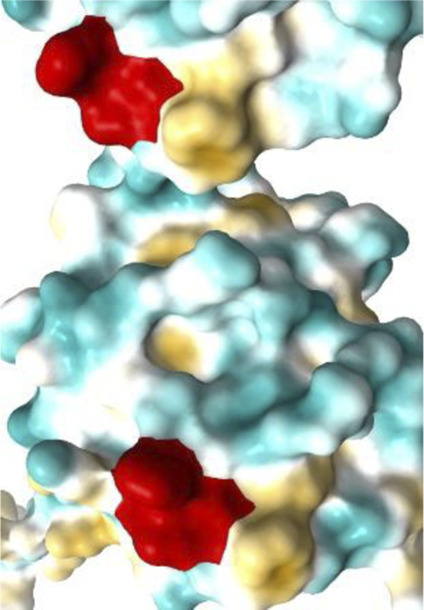

**TABLE 4:** Effects of extracellular PDI on several diseases.

Type of disease	Pathology	Target protein	Effect
Cardiovascular	Thrombosis	Erp57 Alpha-2-b-beta-3-integrin Vitronectin	Impair hemostatic functions Attenuated platelet thrombus Promoted arterial thrombosis
Cardiovascular	Vascular inflammation	Alpha-M-beta-2 integrin L-selectin	Promoted neutrophil recruitment and adhesion Influencing inflammation and tissue injury
Cardiovascular	Myocardial heart attack	PDI	Upregulated in response to ER stress and hypoxia, protects cardiomyocytes from apoptosis
Cardiovascular	Ischemic stroke	PDI	Upregulated in glia and neurons, provides cryoprotection against hypoxia-induced cell death
Cardiovascular	Atherosclerosis	PDI RhoGDI, NOX1	Regulated ROS production and cell migration Involved in vascular smooth muscle cell proliferation and apoptosis
Neurodegenerative diseases	Alzheimer’s Huntington’s disease Amyotrophic lateral sclerosis	PDI	S-nitrosylation of PDI decrease chaperone and isomerase activity Proapoptotic function Activation of unfolded protein response
Infectious diseases	Newcastle disease Cholera Tumorigenesis for polyomavirus (HIV)-1 infection	ERdj5 PDI Erp29 PDI, Erp57 and P5	Increase in viral membrane fusion and access to host cells Displaces the toxic A1 subunit and enables its transfer to cytoplasm Regulation of the mesenchymal-epithelial transition Enhance functional activity of galectin-9 and infectivity
Infertility	N/A	PDI and Erp57	Inhibition of sperm-egg fusion
Lipids homeostasis	Abetalipoproteinemia	PDI	Facilitates the transfer of apolipoprotein to microsomal triglyceride transfer protein
Hemostasis	Thrombus formation	Erp44 Erp57 P5	Adiponectin retention Interaction with calnexin and calreticulin MCH I-related chain modulation
Cancer	N/A	P5 PDI	Facilitates the detachment of tumor -associated proteins, BiP upregulation Activation of the metalloprotease ADAM17
Metabolic disease	Diabetes and obesity	Erp44 PDI	Prevents secretion of adiponectin and regulate inositol phosphate 3 receptor Increase the circulation of apolipoprotein B-containing lipoproteins

It was initially hypothesized that proteins with lower Km values would have a significantly larger hydrophobic surface, featuring larger pockets or a higher number of lipophilic regions. However, our results did not reveal this relationship or any other clear correlation between surface properties and substrate affinity.

The data emphasizes the necessity for further studies to clarify how subtle variations in active site architecture influence PDI catalytic efficiencies and substrate interactions.

Collectively, this multi-layered analysis—integrating primary sequence conservation, secondary structural motifs, and tertiary structural features—highlights a coordinated functional network critical for PDI’s enzymatic activity as oxidoreductases and isomerases. Our findings advance the understanding of structural-functional relationships within PDI proteins, providing a tool kit of structural features that can be combined depending on the target substrate and function, that could potentially have therapeutical application in protein conformational diseases.

### 4.3 Potential of PDI and Dsb in biomedicine

In the context of therapeutic applications, recent research has highlighted the critical roles of) PDI and bacterial Dsb proteins in mitigating diseases associated with protein misfolding. As outlined previously, the DsbA–DsbB oxidative folding system in bacteria provides a mechanistic model for disulfide bond formation (Benham, 2012). This bacterial system provides a model for understanding disulfide bond formation and isomerization that could be mimicked in therapeutic strategies to manage or correct protein misfolding in human diseases (Neves et al., 2017).

Furthermore, PDI, an enzyme located in the endoplasmic reticulum of eukaryotic cells, has shown promise in the treatment of neurodegenerative diseases such as Alzheimer’s and Parkinson’s, which are characterized by protein misfolding and aggregation. As previously described, PDI’s catalytic action supports disulfide rearrangement and prevention of protein misfolding (Hashimoto, 2008).

The exploration of these proteins has led to a deeper understanding of their structural and functional mechanisms, which could be harnessed to develop novel therapeutic interventions. For example, targeting the enzymatic activity of PDI to enhance its isomerase and chaperone functions holds promise for preventing or reversing pathological protein aggregation associated with chronic diseases. This therapeutic potential is underscored by findings in neurodegenerative disorders, where PDI activity is compromised, such as in Alzheimer’s disease, where S-nitrosylation of its active site cysteines impairs its function and contributes to protein misfolding and neuronal degeneration. Beyond Alzheimer’s, PDI dysfunction has been implicated in a range of misfolding diseases, including Parkinson’s disease, amyotrophic lateral sclerosis (ALS), and certain cancers. Under conditions of oxidative stress and ER overload, PDI activity becomes overwhelmed or miss regulated contributing to chronic ER stress and activation of the unfolded protein response (UPR). In neurodegenerative diseases, this can exacerbate protein aggregation and neuronal death. Conversely, in cancer cells, PDI overexpression supports cell survival, redox adaptation, and resistance to apoptosis. These findings highlight the dual role of PDI in health and disease—where restoring its protective function may be therapeutic in degeneration, while inhibiting its pro-survival activity may be advantageous in oncology.

Although PDI is primarily located in the endoplasmic reticulum, fractions of it are present in the general circulation, exerting effects on various organs and tissues (Table 4). The role of extracellular PDI in the triggering of cardiovascular diseases is an avenue of intense research. Thrombosis and vascular inflammation are the target pathologies. The inhibition of the activity of extracellular PDI decreases platelet thrombus formation and generates prolonged tail bleeding times simultaneously (Joly and Swartz, 1994). In diabetes it is related to the activation of platelet alpha-2-b-beta-3-integrin.

The synthesis of this research into Dsb and PDI proteins underscores the potential of these enzymes as targets for the development of new drugs that could manipulate the protein folding process, offering hope for interventions in diseases where misfolding is a key pathology. Such advancements underscore the therapeutic promise of modulating protein folding pathways through targeted interventions in PDI and Dsb enzymatic systems.

### 4.4 Limitations of the study

There is still a long way to go in fully understanding the mechanisms of action and the structure-activity relationship of these proteins. One proposed approach is to conduct enzymatic studies that evaluate a specific activity of different PDI proteins under identical experimental conditions using the same assay. By calculating kcat/Km, each protein could be categorized based on its catalytic efficiency. This data could then be used in a machine learning algorithm to identify patterns among the proteins and apply these findings to a specific therapeutic application.

## Data Availability

The original contributions presented in the study are included in the article/supplementary material or are available in public repositories (PDB), further inquiries can be directed to the corresponding authors.
